# ROS Generation and Antioxidant Defense Systems in Normal and Malignant Cells

**DOI:** 10.1155/2019/6175804

**Published:** 2019-08-05

**Authors:** Anastasiya V. Snezhkina, Anna V. Kudryavtseva, Olga L. Kardymon, Maria V. Savvateeva, Nataliya V. Melnikova, George S. Krasnov, Alexey A. Dmitriev

**Affiliations:** Engelhardt Institute of Molecular Biology, Russian Academy of Sciences, Moscow 119991, Russia

## Abstract

Reactive oxygen species (ROS) are by-products of normal cell activity. They are produced in many cellular compartments and play a major role in signaling pathways. Overproduction of ROS is associated with the development of various human diseases (including cancer, cardiovascular, neurodegenerative, and metabolic disorders), inflammation, and aging. Tumors continuously generate ROS at increased levels that have a dual role in their development. Oxidative stress can promote tumor initiation, progression, and resistance to therapy through DNA damage, leading to the accumulation of mutations and genome instability, as well as reprogramming cell metabolism and signaling. On the contrary, elevated ROS levels can induce tumor cell death. This review covers the current data on the mechanisms of ROS generation and existing antioxidant systems balancing the redox state in mammalian cells that can also be related to tumors.

## 1. Introduction

Reactive oxygen species (ROS) are formed as natural by-products of normal cell activity and participate in cellular signaling [1]. The increase in ROS levels has harmful effects on cell homeostasis, structures, and functions and results in oxidative stress. As such, the disturbance of cellular redox balance is a risk factor for the development of various pathologies [2].

Tumor cells are characterized by a high level of ROS. ROS overproduction can result from changes in many processes, such as oxidative phosphorylation (OXPHOS), transition metal ions, oxidase activity, protein folding, thymidine, and polyamine catabolism [3–7]. ROS can be generated both in various cellular compartments and in the tumor microenvironment.

ROS have a dual role in cancer development; on one hand, they can promote molecular genetic alterations that are necessary for tumor initiation, growth, and progression, as well as acquisition of treatment resistance [8]. On the other hand, permanent elevated ROS levels have cytotoxic effects, inducing activation of apoptotic pathways or inhibiting resistance to anticancer treatments [9].

In this review, we discuss the main sources of ROS production in animal cells and the antioxidant defense systems that could be implicated in the redox state of cancer cells (to a significant or less significant extent).

## 2. Sources of ROS Generation and Antioxidant Defense Systems

### 2.1. Mitochondria

Mitochondria are a prime source of endogenous ROS due to its main role in oxidative ATP production, in which molecular oxygen (O_2_) is reduced to water in the electron transport chain. The superoxide radical (O_2_^•−^) is produced at a number of sites in the mitochondria, including complex I (sites IQ and IF), complex III (site IIIQo), glycerol 3-phosphate dehydrogenase, Q oxidoreductase, pyruvate dehydrogenase, and 2-oxoglutarate dehydrogenase [10]. All the sites release superoxide radical into the mitochondrial matrix (MM), and two of them, complex III (site IIIQo) and glycerol 3-phosphate dehydrogenase, also generate ROS into the intermembrane mitochondrial space (IMS). Manganese superoxide dismutase (Mn-SOD) converts the superoxide radical to hydrogen peroxide (H_2_O_2_) in the MM, while Cu- and Zn-SOD convert the superoxide radical in the IMS or cytosol [11]. The H_2_O_2_ in the MM can further be converted by mitochondrial aconitase to a hydroxyl radical (^•^OH) via a Fenton reaction [12]. One more site of ROS production in the mitochondria is the cytochrome (CYP) catalytic cycle. CYP enzymes metabolize a wide range of organic substrates (lipids, steroid hormones, xenobiotics, and others) to give rise to superoxide radical and H_2_O_2_ as by-products [13]. Several CYP family members were shown to be present in the mitochondrial membrane of steroidogenic organs, as well as in the liver and kidney [14]. Furthermore, several other mammalian proteins, such as NADH-cytochrome *b5* reductase [15], dihydroorotate dehydrogenase [16, 17], complex II (succinate dehydrogenase) [18], and monoamine oxidases (MAO) [19], were shown to generate ROS in the mitochondria.

Mitochondria are protected from ROS by multiple defense systems and antioxidants: glutathione peroxidases (GPXs), thioredoxin peroxidases (TRXPs), superoxide dismutases (SODs), peroxiredoxins (PRDXs), glutathione (GSH), thioredoxin 2 (TRX2), glutaredoxin 2 (GRX2), cytochrome c oxidase (complex IV), coenzyme Q, ascorbic acid, tocopherol, vitamin E, and carotene [20–26]. Moreover, catalase (CAT), which commonly detoxifies H_2_O_2_ in the peroxisome, was found in rat heart mitochondria (but not in other tissues) [27, 28].

Mitochondrion-generated ROS were widely shown to be implicated in various human pathologies, including inflammation, cancer, mitochondrial and neurodegenerative diseases, diabetes, chronic diseases, and aging [3, 29–32]. Elevated ROS levels and mitochondrial dysfunction, present in many cancers, lead to oxidative damage of cellular structures, in particular, genomic and mitochondrial DNA, somatic mutations, genome instability, activation of oncogenes and inactivation of tumor suppressor genes, and alterations in metabolic and signaling pathways with simultaneous activation of compensatory antioxidant mechanisms, that all contribute to cell transformation [33]. However, overproduction of ROS can also promote tumor cell apoptosis and such strategies have effectively been used for anticancer treatment [34].

### 2.2. Transition Metal Ions

One of the major mechanisms of metal carcinogenicity is the ability of transition metal ions to induce oxidative stress [35]. Fenton and Haber-Weiss reactions are frequently responsible for ROS generation in living cells [36]. During these reactions, H_2_O_2_ is decomposed with the participation of metal ions, such as iron (Fe), copper (Cu), zinc (Zn), and aluminum (Al), leading to the production of hydroxyl radical (^•^OH) and hydroxyl anion (OH^−^) [37]. Other carcinogenic metal ions (antimony, arsenic, chromium, cobalt, nickel, and vanadium) were also supposed to be able to generate ROS in cellular redox reactions [36]. The hydroxyl radicals produced can attack the DNA, causing oxidative DNA adduct formation. The adducts 8-hydroxy-2′-deoxyguanosine (8-OHdG) and 8-oxo-7,8-dihydro-2′-deoxyguanosine (8-oxodG) are the most predominant, resulting from the addition of hydroxyl radicals to guanine. These compounds are widely considered as markers of endogenous oxidative DNA damage as well as a risk factor for cancer development [38].

### 2.3. Peroxisome Activity

Peroxisomes have multiple functions in living cells, including fatty acid *β*-oxidation and *α*-oxidation as well as metabolism of purines, polyamines, amino acids, glyoxylate, reactive oxygen and nitrogen species (RNS), transition metal ions, and others [39, 40]. Peroxisomes generate a wide range of ROS and RNS: H_2_O_2_, superoxide radical (O_2_^•−^), hydroxyl radical (^•^OH), nitric oxide (NO^•^), and peroxynitrite (ONOO^−^) [41].

The peroxisome was first described as an H_2_O_2_-producing and H_2_O_2_-degrading organelle [42]. Despite the presence of CAT, peroxisomes are one of the main sources of H_2_O_2_ [43, 44]. H_2_O_2_ is released as a by-product during the normal catalytic activity of many peroxisomal enzymes, and it can also be generated by the spontaneous or enzymatic dismutation of the superoxide radical [45]. H_2_O_2_ can generate hydroxyl radicals (^•^OH) via a Fenton reaction [46].

Peroxisomes produce superoxide radicals (O_2_^•−^) in both the matrix and membrane. In the matrix, two enzymes are responsible for O_2_^•−^ generation, xanthine oxidoreductase (XOR), and urate oxidase (UO) [47, 48]. XOR catalyzes the formation of uric acid during purine metabolism that is further converted to allantoin by UO. Both enzymes generate O_2_^•−^ and H_2_O_2_. However, UO expression was not detected in humans and some primates indicating that uric acid is a terminal compound of purine metabolism [49]. The other source of superoxide radicals is an electron transport chain in the peroxisomal membrane [50]. Moreover, XOR also catalyzes the reduction of nitrates and nitrites to nitric oxide (NO^•^) [51]. NO^•^ can also be produced from L-arginine, in a reaction catalyzed by nitric oxide synthase (NOS) [52]. The reaction of O_2_^•−^ with NO^•^ results in a highly reactive compound called peroxynitrite (ONOO^−^) [53].

Several antioxidant systems regulate ROS levels in peroxisomes and defend cells from oxidative damage. These include a number of enzymes, such as CAT, superoxide dismutases, peroxiredoxins, glutathione S-transferases (GST), and epoxide hydrolase 2 (EPHX2), as well as nonenzymatic low-molecular weight antioxidants, which are reviewed in detail elsewhere [39, 45, 50].

Changes in redox homeostasis contribute to cancer development and progression [54]. The peroxisome maintains cellular oxidative balance, and dysregulation of its activity is associated with carcinogenesis. Thus, reduced CAT activity leads to ROS generation and oxidative stress resulting in DNA damage and genome instability promoting cancer development. Decreased CAT expression has been shown in hepatocellular carcinoma and colon, lung, kidney, and prostate cancers, as well as in precancer states, such as prostatic intraepithelial neoplasia (PIN) and cervical intraepithelial neoplasia (CIN) [55–60]. In several cases, decreased CAT activity was associated with a reduced number of peroxisomes [57, 61].

### 2.4. Endoplasmic Reticulum

The endoplasmic reticulum (ER) has many general cellular functions, such as protein folding, synthesis, transport, and posttranslational modifications, as well as lipid metabolism and calcium storage [62]. Alterations in the folding pathway lead to accumulation of misfolded and unfolded proteins in the ER lumen resulting in ER stress. This disrupts cell homeostasis and initiates the unfolded protein response (UPR) [63, 64]. UPR triggers ROS production, and ROS, in turn, can promote ER stress [65, 66]. In tumor cells, the UPR signaling pathway serves as an adaptive to the stress mechanism supporting their survival and propagation [67]. However, if ER stress is prolonged, the UPR triggers tumor cell apoptosis [68].

Oxidative protein folding gives rise to a highly oxidative environment in the ER lumen [69]. Protein disulfide isomerase (PDI) catalyzes thiol-disulfide exchange reactions, which form a native disulfide bond in proteins [70]. During this process, PDI is oxidized by endoplasmic reticulum oxidoreductin-1 (ERO1), which accepts electrons from a reduced PDI and transfers them to molecular oxygen, thereby generating H_2_O_2_ [69, 71]. ERO1 also catalyzes the conversion of glutathione (GSH) to glutathione disulfide (GSSG), besides PDI oxidation. Accumulation of both H_2_O_2_ and oxidized glutathione causes ER stress. Furthermore, the ratio between GSH and GSSG is an essential marker of the redox status in the ER lumen. Moreover, GSH was proposed as a potential protection mechanism from ER-associated ROS damage [72]. PDI and ERO1 were found to be upregulated in different types of cancer and were implicated in cancer progression and metastasis. PDI and ERO1 overexpression has been found in patients with non-small cell lung cancer (NSCLC) and was significantly associated with shorter overall survival [73]. Expression of several members of the PDI family was upregulated in ovarian and colorectal cancers [74, 75] while ERO1 overexpression was correlated with the progression and metastasis of breast cancer, as well as with poor survival and high recurrence rates in gastric cancer [76–78]. ERO1 knockout led to the reduced growth of colorectal cancer cells under hypoxic conditions [79]. Elevated ERO1 expression was associated with poor prognosis in cervical cancer [80]. In the same study, ERO1 knockout inhibited tumor growth and migration of cervical cancer cells.

PDI is also involved in H_2_O_2_ generation through interconnections with NOX1 and NOX4, which belong to the nicotinamide adenine dinucleotide phosphate (NADPH) oxidase protein family [81, 82]. In addition, NOX4 occurs in the NOX4-p22^phox^ complex in the ER membrane and is involved in releasing H_2_O_2_ in the ER lumen [83]. One more site of ROS generation in the ER is the microsomal monooxygenase (MMO) system. This is a multienzyme system consisting of multiple cytochrome P450 species, NADPH-P450 reductase (NPR), and cytochrome *b_5_* [84]. MMO catalyzes the oxygenation of hydrophobic exogenous compounds and some endogenous substrates, resulting in a production of superoxide radicals and H_2_O_2_ [85, 86]. Increased MMO-derived ROS production and greater rates of microsomal lipid peroxidation were shown to be associated with the activation of cytochrome P450 2E1 (CYP2E1) [87, 88].

### 2.5. Thymidine Catabolism

Thymidine phosphorylase (TP) is a rate-limiting enzyme in thymidine catabolism that catalyzes the reversible conversion of thymidine to thymine and 2-deoxy-D-ribose-1-phosphate (DR1P) [89]. TP is upregulated in many tumors and plays an important role in angiogenesis, apoptosis evasion, invasion, and metastasis, as well as in chemotherapy response [90]. Recently, Tabata and coauthors have revealed that TP activity increases NADPH levels via the pentose phosphate pathway (PPP) which activates NADPH oxidase-dependent production of ROS in cancer cells [6, 91]. Previously, it was shown that the addition of thymidine to a TP-overexpressing bladder carcinoma cell line induces cellular oxidative stress [92]. The authors proposed another potential mechanism of TP-induced ROS production. This mechanism is based on excess 2dDR1P produced during thymidine phosphorylation that could be further subjected to transition metal-catalyzed oxidation, leading to ROS generation.

### 2.6. Polyamine Catabolism

The natural polyamines (PAs), putrescine, spermidine, and spermine, are involved in multiple basic cellular functions, including growth, proliferation, differentiation, apoptosis, migration, and protection from stresses. They are involved in protein posttranslational modifications, regulation of ion channels, maintenance of nucleic acid, and protein structure and stability, as well as cell-cell communications [93]. PA content and catabolism are strongly regulated at different levels by key enzymes in the biosynthesis and transport systems [94]. However, dysregulation of PA catabolism is frequently observed in cancer [95].

In mammals, putrescine is the first polyamine of the PA catabolism. It is synthesized with the participation of ornithine decarboxylase (ODC). Putrescine is further converted to higher polyamines, spermidine and spermine, a reaction catalyzed by spermidine synthase (SPDS) and spermine synthase (SPMS), respectively. S-Adenosylmethionine decarboxylase (AdoMetDC) supplies the aminopropyl groups in these reactions. The activities of ODC and AdoMetDC are considered to be a rate-limiting factors of PA biosynthesis. The other branch of PA catabolism involves the interconversion cycles where spermine is degraded to spermidine and spermidine to putrescine with the generation of toxic-reactive aldehydes and ROS. Spermine oxidase (SMO) catalyzes the conversion of spermine to spermidine, which is accompanied by 3-aminopropanal and H_2_O_2_ release. Spermidine and spermine can also be converted to prior polyamines, with the help of spermidine/spermine-N1-acetyltransferase (SSAT) and acetylpolyamine oxidase (APAO/PAOX). N-Acetyl-3-aminopropanaldehyde and H_2_O_2_ are produced as by-products of these reactions [94, 96]. Additionally, another degradative enzyme related to PA catabolism is a diamine oxidase (DAO) that oxidizes putrescine to H_2_O_2_, ammonia, and 4-aminobutanal. However, as putrescine is present in relatively low amounts in most mammalian tissues, this reaction does not generate significant amounts of ROS [97].

Increased PA catabolism can lead to an elevated level of ROS and oxidative stress. Overexpression of *SMOX* and *SAT1*, which encode the SMO and SSAT enzymes, respectively, was shown to be interconnected with infection, inflammation, and high risk of cancer. Several studies have reported that bacterial cytotoxins upregulate the expression of *SMOX*. The *Helicobacter pylori* virulence factor, cytotoxin-associated gene A (CagA) protein, promotes an increase in the *SMOX* mRNA level and enzyme activity in both human and animal gastric epithelial cells. This results in a significant increase in extra- and intracellular H_2_O_2_, leading to DNA damage and apoptosis, which can further contribute to inflammation and carcinogenesis [98, 99]. Using a gerbil model, it was shown that *H. pylori* infection induces overexpression of SMO and oxidative DNA damage and is associated with a high risk of gastric dysplasia and adenocarcinoma [100]. Furthermore, SMO expression was increased in gastric cancer patients infected with *H. pylori* [101]. In the same study, it was reported that activation of EGFR and ERBB2 signaling is involved in *H. pylori*-induced upregulation of *SMOX*.

Enterotoxigenic *Bacteroides fragilis* (ETBF) infection results in chronic inflammation and can promote colorectal carcinogenesis [102]. *B. fragilis* toxin (BFT) has also been reported to increase *SMOX* expression leading to ROS generation and DNA damage in colonic epithelial cells [103]. A study involving patients with colorectal cancer has revealed that *SMOX* overexpression could be caused by the activation of the transcription factor C/EBP*β*, which is involved in the regulation of inflammation and immunity, rather than the ETBF infection [104]. The association of increased *SMOX* expression and chronic inflammation was also observed in several precancerous conditions such as prostatic intraepithelial neoplasia (PIN) and chronic hepatitis [105–107]. Interestingly, drug-induced modulation of polyamine catabolism in hepatic cells results in them undergoing an epithelial-mesenchymal transition- (EMT-) like dedifferentiation, which is not, however, associated with elevated ROS levels [108]. This can indicate that ROS overproduction caused by increased polyamine catabolism together with chronic inflammation could be a precursor event of cancer development, but not tumor progression (with respect to metastasis).

On the contrary, an antioxidant role of polyamines has been proposed. Multiple protection mechanisms from oxidative damage with PA participation were reported: direct ROS scavenging [109, 110], changes in DNA structure and conformation which reduce the possibility of its interactions with reactive species [109, 111–114], formation of chelates with metals at low concentration which prevents ROS generation, particularly hydroxyl radicals [115], or a combination of these mechanisms. Additionally, PA metabolism was linked to p53-mediated ferroptosis in response to oxidative stress [116]. This mechanism is based on p53-induced SSAT activation, in the presence of high levels of ROS, leading to downstream modulation of the expression of ferroptosis components.

### 2.7. Oxidase Activity

Diamine oxidase (DAO) is a copper-containing amine oxidase that catalyzes the oxidation of polyamines, such as histamine, putrescine, spermidine, and to a lesser extent spermine [97, 117]. All these reactions generate H_2_O_2_. DAO activity in mammals varies and was found to be tissue specific. As such, high DAO activity was found in the placenta, kidneys, lungs, small intestine, and liver [118, 119]. Moreover, elevated DAO activity was found in prostate, breast, ovarian, cervical, and endometrial cancers [120–122] and a decrease in activity was found in colorectal cancer [123]. Increased plasma/serum DAO activity was detected in patients with endometrial, lung, and thyroid cancers [124–126]. Moreover, serum/plasma DAO activity has been proposed as an indicator of mucosal injury during chemotherapy and can be used for monitoring anticancer drug toxicity [127, 128].

Acetylpolyamine oxidase (APAO/PAOX) is related to flavin adenine dinucleotide- (FAD-) containing enzymes and catalyzes the oxidation of both spermidine and spermine in peroxisomes. Depending on the substrate, N1-acetylspermine or N1-acetylspermidine, APAO produces spermidine or putrescine, respectively, generating H_2_O_2_ as a by-product [129]. The presence of starting substrates of APAO is controlled by SSAT activity [130]. Induction of the SSAT/APAO pathway can increase oxidative stress; however, it seems to have a more significant contribution than the one-step spermine oxidation reaction catalyzed by SMO [131].

Spermine oxidase (SMO) is a FAD-dependent oxidase that acts directly on spermine generating spermidine, H_2_O_2_, and 3-aminopropanal [132, 133]. Unlike APAO, SMO is not a peroxisomal enzyme and is located in cytoplasm or nucleus [134]. SMO activity can produce higher levels of oxidative cellular damage [135]. Elevated expression of *SMOX* was shown in prostate and colorectal cancers [104, 105]. Furthermore, *SMOX* overexpression followed by downstream oxidative damage, chronic inflammation, and carcinogenesis is often induced by infections (discussed above).

Xanthine oxidoreductase (XOR) is a molybdenum iron-sulfur flavin hydroxylase that exists in two forms, xanthine dehydrogenase (XDH) and xanthine oxidase (XOD). XDH can be converted to XOD either irreversibly by proteolysis or reversibly by modification of cysteine residues [136, 137]. The enzyme catalyzes the oxidation of hypoxanthine to xanthine or xanthine to uric acid during purine metabolism [138]. Both XDH and XOD generate H_2_O_2_ and O_2_^•−^ through NADH oxidation [139]. Nevertheless, for XDH, NAD+ is a more preferable substrate compared to oxygen, and therefore, it cannot directly produce ROS [140]. XOR-released superoxide radicals rapidly react with nitric oxide (NO^•^) generating peroxynitrite (ONOO^−^). NO^•^, in turn, is produced by NOS activity or even by XOR under hypoxic conditions [141]. The association of XOR with neoplastic transformation was first reported many years ago [142]. Decreased XOR activity was frequently found in many animal and human tumors [143]. XOR activity was increased in meningioma, astrocytoma, and laryngeal and colorectal cancers [144–146]. This indicates that dysregulation of purine metabolism and ROS levels can play a role in tumor pathogenesis.

Cytochrome P450 (CYP) oxidase is part of the microsomal electron transport system. It belongs to the CYP superfamily of integral membrane proteins that catalyze the oxidation of numerous organic substrates, accompanied by the reduction of molecular oxygen [147]. CYP enzymes also have peroxygenase and peroxidase activity, using H_2_O_2_ either for direct oxidation of substrates or as a donor of oxygen atoms [148]. The prosthetic heme group in CYP enzymes is essential for their activity [149]. H_2_O_2_ and superoxide radicals are produced during the CYP monooxygenase cycle; the former can be further decomposed to hydroxyl radicals (^•^OH) in the presence of ferrous iron via a Fenton reaction [150]. CYP enzymes are predominantly located in the endoplasmic reticulum and mitochondria and are expressed in many mammalian tissues [151]. CYP-related ROS generation depends on CYP isoforms, content and type of substrates, pH, ionic strength, the action of cytochrome *b5*, oxygen concentration, oligomerization, and so on [150, 152, 153]. Studies have shown that CYP2E1 induces higher ROS production than other CYP enzymes and that its activation or overexpression leads to increased ROS levels [152, 154]. CYP enzyme activity can promote carcinogenesis through increased ROS production. As such, overexpression of CYP family genes was observed in many cancers [150].

The NADPH oxidase (NOX) family includes seven members: NOX1, NOX2, NOX3, NOX4, NOX5, dual oxidase 1 (DUOX1), and DUOX2 [155]. These are transmembrane proteins that transfer an electron from the NADPH substrate to FAD across biological membranes in order to reduce oxygen to a superoxide radical [156]. Dysregulation of NOX activity leads to elevated ROS production that can contribute to tumorigenesis, cell transformation, tumor growth, angiogenesis, and metastasis. NOX-derived ROS were shown to be implicated in many common cancer types (e.g., bladder, colorectal, breast, kidney, lung, and prostate cancers), as well as acute myeloid leukemia and non-Hodgkin's lymphoma [157–161]. Moreover, NOX family members are involved in host defense and innate immunity. NOX could be activated by various infectious agents resulting in increased ROS production which promotes the death of the infected cells as well as an inflammatory response [162]. Toll-like receptors (TLRs) located at the plasma membrane recognize conserved structures of bacteria, viruses, or fungi and trigger downstream signaling. TLRs interact with NOX, leading to increased ROS generation, as well as subsequent activation of the transcription factor NF-*κ*B, and production of cytokines and chemokines [163–165]. Nucleotide-binding oligomerization domain- (NOD-) like receptors (NLRs) can also recognize components of the bacterial cell wall and other bacterial molecules (e.g., RNA, toxins, and ligands). NLR family members, NACHT lectin-like receptor and PYD domain-containing proteins (NLRPs), promote the assembly of inflammasomes and mediate the activation of inflammatory caspases [166]. ROS were shown to be required for the activation of NLRP3-mediated inflammasomes, and NOX, as one of the main sources of ROS production, can be involved in NLR signaling [167]. The important role of NOX-produced ROS in the NLR system was also reported by Lipinski et al. [168]. Additionally, hepatitis C virus (HCV) infection can induce NOX1 and NOX4 expression, leading to increased ROS generation in hepatocytes [169]. It was shown that HCV modulates NOX4 through TGF-beta [169, 170]. Moreover, HCV can induce immune dysfunction via HCV nonstructural protein 3- (NS3-) induced release of ROS from phagocytes, resulting in the apoptosis of T lymphocytes, natural killer (NK) cells, and NKT cells [171, 172].

Cyclooxygenases (COX) and lipoxygenases (LOX) participate in polyunsaturated fatty acid (PUFA) metabolism, by producing bioactive eicosanoids. COX and LOX oxygenate arachidonic acid resulting in the formation of prostaglandin G2 and H2 (PGG2/PGH2) and fatty acid hydroperoxide, respectively, a reaction that is accompanied by ROS release [173]. Peroxyl radicals (ROO^•^) and alkoxyl radicals (RO^•^) are intermediates of the hydroperoxide metabolism. Moreover, the arachidonic acid pathway itself could be responsible for ROS generation via NADPH oxidase activation [174]. In spite of the similarity between COX/LOX-catalyzing reactions, they have a completely different sequence, structure, cellular localization, and tissue expression [175]. COX is present in three isoforms - constitutive (COX-1), inducible (COX-2), and splice variant of COX-1 (COX-3) [176]. The *PTGS1* gene encodes for both COX-1 and COX-3 isoenzymes; however, COX-3 activity in humans remains unclear [177, 178]. *PTGS2* encodes for COX-2. COX-1 predominantly regulates basal prostaglandin metabolism and normal tissue homeostasis [179]. COX-2 is induced by proinflammatory stimuli, cytokines, and growth factors in response to inflammation, tissue injury, and tumorigenesis [180, 181]. COX-2 overexpression and increased prostaglandin biosynthesis have been found in both the precancer stages and various tumors [173, 182–184]. COX1 was upregulated in breast, cervical, ovarian, endometrial, and colorectal cancers, as well as in cholangiocarcinoma [185–190].

LOX constitutes a large family of nonheme iron-containing dioxygenases; five LOX isoenzymes were found in humans (5-LOX, 12-LOX, 12R-LOX, two 15-LOX, and 3-LOX/E-LOX). Dysregulated LOX expression was observed in various tumors and animal models. A dual role of LOX in tumorigenesis has been proposed, as they can be involved in both neoplastic transformation and tumor suppression [173].

Monoamine oxidases A and B (MAOA and MAOB) are mitochondrial FAD-dependent enzymes encoded by separate genes [191, 192]. They catalyze the oxidative deamination of monoamines, including monoamine neurotransmitters (e.g., norepinephrine, dopamine, serotonin, and epinephrine), exogenous dietary amines, and drugs, generating H_2_O_2_ and aldehydes as by-products [193, 194]. Excessive MAO activity leads to enhanced ROS production and mitochondrial damage and is implicated in aging, cardiovascular diseases, neurodegenerative disorders, and cancer [195–197]. Increased MAOA expression was found in lung, prostate, and breast cancers, as well as hepatocellular carcinoma and cholangiocarcinoma [198–203]. Moreover, several studies suggested that MAOA can promote cancer progression through induction of EMT [197, 198]. Conversely, MAOA was downregulated in a number of cancer tissues and significantly differed between them [204]. In this case, the potential mechanism of tumor progression can be related to an increase in the amounts of MAOA substrate epinephrine [205, 206].

Lysyl oxidase is a family of copper-dependent enzymes that play a primary role in the remodeling of the extracellular matrix (ECM). They catalyze the conversion of specific lysine residues into reactive aldehyde groups in collagen and elastin, forming protein crosslinks [207]. The lysyl oxidase catalytic cycle also produces H_2_O_2_, which can increase oxidative stress thereby promoting carcinogenesis. Overexpression of lysyl oxidase and its involvement in tumor progression and metastasis were shown in various cancers [208–214].

### 2.8. Signaling Pathways

The PI3K/AKT/PTEN signaling pathway is implicated in NOX-derived ROS production [215]. PTEN (phosphatase and tensin homolog) is a tumor suppressor responsible for the negative regulation of PI3K/AKT signaling. Loss of *PTEN* expression or mutations in the gene, as well as dysregulation of the PI3K/AKT signaling pathway, were frequently found in many tumors [216, 217].

NOXs are multimeric enzymes consisting of several proteins that are distributed between the membrane and cytosol when inactive. Upon activation by different stimuli, the cytosolic subunits interact with integral membrane subunits, forming the functional NOX enzymes which can generate ROS [218]. It was shown that PI3K/AKT inhibitors can reduce NOX-dependent ROS generation through the inhibition of NOX subunit translocation into the membrane. In addition, membrane depolarization with downstream PI3K/AKT and (protein kinase C) PKC activation causes NOX assembly and ROS production [215]. Moreover, oxidative stress inhibits PTEN-induced PI3K/AKT signaling, which promotes both the expression of cell survival genes and ROS production [219]. Such mechanisms may contribute to tumor cell proliferation and growth under oxidative conditions. Several other enzymes, including PKC, mitogen-activated protein kinases (MAPK), cAMP-dependent protein kinases (PKA), p21-activated kinases (PAK), and PKB/AKT, can modulate the activation of NOXs through phosphorylation of their cytosolic subunits, thereby increasing the level of ROS [220]. All the aforementioned enzymes were shown to be greatly involved in cancer development [221–223].

Transcription factor p53 is a widely known tumor suppressor, involved in regulating the expression of various genes encoding for both ROS-producing and antioxidant-related components [224]. p53 induces the expression of glutathione peroxidase 1 (﻿GPX1) and mitochondrial superoxide dismutase 2, both components of the key antioxidant defense system [225, 226]. Moreover, p53 regulates the expression of sestrins (*SESN1* and *SESN2*) that are required for peroxiredoxins regeneration [227]. Phosphate-activated mitochondrial glutaminase (GLS2) converts glutamine to glutamate, which is a precursor for glutathione synthesis. GLS2 expression can be induced by p53 in response to DNA damage or oxidative stress to promote antioxidant defense by controlling the GSH/GSSG ratio [228]. Other p53-inducible antioxidant genes are *TIGAR* and *ALDH4*. TIGAR negatively regulates glycolysis and decreases intracellular ROS levels [229], while aldehyde dehydrogenase 4 (ALDH4) is a NAD+-dependent enzyme that catalyzes the second step of the proline degradation pathway in the mitochondrial matrix [230]. Overexpression of *ALDH4* in p53-null cells inhibits ROS generation and apoptosis [231].

The function of prooxidant p53 is based on its ability to regulate the expression of genes encoding for prooxidant enzymes, such as PUMA (the p53-upregulated modulator of apoptosis), p66Shc (66 kDa Src collagen homologue (Shc) adaptor protein), and other proteins encoded by a group of p53-induced genes (PIGs). PUMA overexpression induces BCL2-associated X (BAX) protein-dependent ROS generation (predominantly superoxide radicals and H_2_O_2_) and apoptosis in colorectal cancer cells [232]. p66Shc generates mitochondrial H_2_O_2_ as signaling molecules for the induction of apoptosis [233]. PIG is a family of proteins, whose several members were shown to influence the cell redox status. P53-induced activation of PIGs results in increased ROS levels and mitochondria-derived apoptosis in colorectal cancer cells [234]. It was shown that p53 promotes the expression of *PIG3*, *BAX*, and *PUMA* leading to an increase in intracellular ROS levels and induction of apoptosis. Moreover, the authors have demonstrated that p53 induction is associated with the excessive ROS release by mitochondria, which supports its prooxidant role [235].

## 3. Role of Glycolysis and the Pentose Phosphate Pathway in Antioxidant Defense

Glycolysis and the pentose phosphate pathway are involved in ROS detoxification. Glycolysis occurs in living cells both in anaerobic and aerobic conditions. Aerobic glycolysis generates pyruvate that is converted to acetyl-CoA with the release of carbon dioxide in the mitochondrial tricarboxylic acid (TCA) cycle, while in the absence of oxygen, lactate is produced [236]. Most tumor cells use anaerobic glycolysis even in the presence of oxygen; this phenomenon is termed the “Warburg effect” [237]. Glycolysis reprogramming allows tumor cells to redirect this process to support *de novo* nucleotide synthesis during proliferation [238]. Dysregulation in the expression of genes encoding for key glycolytic components has been found in many tumors [239–246]. Increased glycolysis in tumor cells can reduce ROS production via a decrease in OXPHOS activity [247].

The NADPH/NADP+ ratio is important for antioxidant defense; NADPH acts as a donor of reductive potential to glutathione and thioredoxin reductases. The main source of NADPH is the oxidative branch of the pentose phosphate pathway (ox-PPP) [248]. Glucose-6-phosphate (G6P) derived from glucose phosphorylation by hexokinases (HKs) is reduced to 6-phosphogluconate and NADPH via glucose-6-phosphate dehydrogenase (G6PD) during the first step of ox-PPP. In the next step, 6-phosphogluconate dehydrogenase (6PG) catalyzes the oxidative decarboxylation of 6-phosphogluconate to ribulose-5-phosphate (Ru5P) providing the additional NADPH [249]. The NADP+/NADPH ratio regulates G6PD and 6PG activity in order to produce more NADPH for oxidative stress prevention [250]. Several glycolytic enzymes, such as phosphofructokinase-1 (PFK1), glyceraldehyde 3-phosphate dehydrogenase (GAPDH), and pyruvate kinase (PK), as well as the TP53-inducible glycolysis and apoptosis regulator (TIGAR), are involved in the redirection of glycolytic flux through the ox-PPP in order to reduce the ROS level [251]. Moreover, the acceleration of glycolysis and PPP in a tumor cell can protect it from oxidative damage [252].

## 4. Tumor Microenvironment

Solid tumors are commonly infiltrated with different types of cells, including cancer-associated fibroblasts (CAFs), immune cells, pericytes, adipocytes, and other tissue-associated cells. This forms a distinct tumor microenvironment that comprises cell-cell and cell-extracellular matrix interactions, as well as many soluble factors [253, 254]. The latter include vascular endothelial growth factors (VEGFs), fibroblast growth factors (FGFs), angiopoietins (ANGs), transforming growth factors (TNFs), ROS, chemokines, cytokines, exosomes, microRNAs, Ca^2+^, K^+^, Na^+^, H^+^, and other ions [255–257]. The tumor microenvironment plays an essential role in tumor initiation, progression, and metastasis [258]. It is also involved in the resistance to targeted therapy, radiation, and chemotherapy, as well as sensitivity to immunotherapy [259, 260].

Relatively stable ROS, such as H_2_O_2_, which is produced at a high level by tumor cells, can diffuse into the extracellular space. H_2_O_2_ can freely cross membranes; however, the cells seem to regulate H_2_O_2_ transport by changes in membrane lipid composition, thereby maintaining cellular H_2_O_2_ concentration [261]. Aquaporins (AQPs) have also been found to be transporters of H_2_O_2_ [262, 263]. Moreover, superoxide dismutase 3 (SOD3, EC-SOD) and NADPH oxidase provide extracellular ROS sources. SOD3, which is located in the extracellular space, catalyzes the dismutation of the superoxide anion into H_2_O_2_ [264], while several NOX isoforms generate H_2_O_2_ and O_2_^•−^ outside cells [156, 265]. Extracellular ROS signaling in tumor cells with the participation of SOD and NOX enzymes has been well described by Bauer et al. [266]. Briefly, NOX located in the plasma membrane produces O_2_^•−^ into the extracellular space. The superoxide radical, in turn, dismutates to H_2_O_2_ during the hypochlorous acid (HOCl) pathway. Peroxidases (POD) use the H_2_O_2_ as a substrate to generate HOCl, which further reacts with hydroxyl anions (OH^−^) leading to the formation of hydroxyl radicals (^•^OH). NOX-generated O_2_^•−^ is reduced to H_2_O_2_ either by SOD3 or spontaneously; membrane CAT decomposes the produced H_2_O_2_ thereby inhibiting HOCl signaling. CAT can also decompose peroxynitrite (ONOO^−^) derived from the reaction between NO and H_2_O_2_ that prevents hydroxyl radical formation through the NO/peroxynitrite pathway.

Apart from tumor cells, cancer-associated fibroblasts also release extracellular H_2_O_2_ that induces oxidative stress in normal fibroblasts, triggering their reprogramming to CAFs and promoting field cancerization, epithelial cell transformation and growth, and cancer aggressiveness [267]. Immune cells, such as myeloid-derived suppressor cells (MDSCs), tumor-associated macrophages (TAMs), regulatory T cells (Tregs), neutrophils, eosinophils, and mononuclear phagocytes, can also generate ROS (mainly H_2_O_2_) into the tumor microenvironment [253, 268].

**Figure 1:**
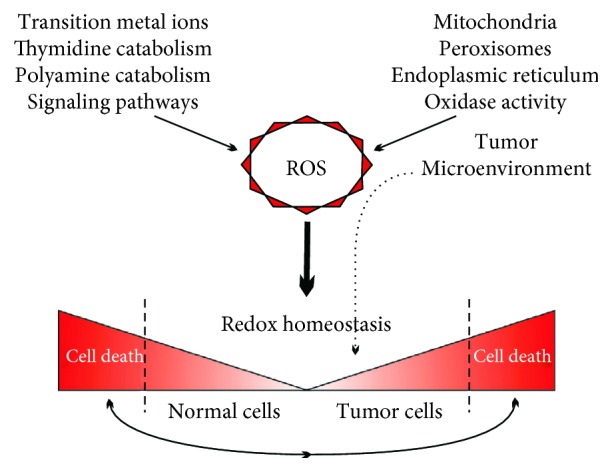
Main sources of ROS production in normal and tumor cells.

The major mechanisms of ROS generation and detoxification are presented in Table 1 and Figure 1.

## 5. Conclusions

ROS are generated by multiple cellular processes and can be overproduced in response to different stimuli. Normal cells can maintain oxidative homeostasis owing to the activity of various antioxidant systems which control ROS production through changes in metabolic and signaling pathways. Upon a permanent increase in ROS levels, the antioxidant defense mechanisms can promote cell death. However, oxidative stress damages many molecules, cell structures, and functions leading to the development of pathological states, such as inflammation, aging, neurodegenerative disorders, and cancer. ROS are greatly implicated in tumorigenesis, and summarizing the current data on ROS biology is important for understanding the mechanisms of tumor initiation, promotion, and progression, as well as for treatment development.

## Figures and Tables

**Table 1:** ROS and major mechanisms of their generation and detoxification.

ROS	Generation	Detoxification
Superoxide radical (O_2_^•−^)	Mitochondrial respiratory chain Electron transport chain in the peroxisomal membrane Superoxide dismutases CYP catalytic cycle Mitochondrial enzymes (glycerol 3-phosphate dehydrogenase, 2-oxoglutarate dehydrogenase, NADH-cytochrome b5 reductase, etc.) Xanthine oxidoreductase	Superoxide dismutases Polyamines

Hydrogen peroxide (H_2_O_2_)	Spontaneous dismutation of superoxide radicals Polyamine catabolism Thymidine catabolism NADPH oxidases Monoamine oxidases Lysyl oxidases Dihydroorotate dehydrogenase CYP catalytic cycle Peroxisomal enzymes (acyl-CoA oxidases, d-amino acid oxidase, d-aspartate oxidase, etc.) Microsomal monooxygenase (MMO) system Normal protein folding/unfolded protein response Polyunsaturated fatty acid metabolism	Polyamines Glutathione peroxidases Thioredoxin peroxidases Catalase Peroxiredoxins Glutathione S-transferases Glutaredoxins Thioredoxins Nonenzymatic scavengers^∗^ Glycolysis Pentose phosphate pathway

Hydroxyl radical (^•^OH)	Fenton and Haber-Weiss reactions Thymidine catabolism (supposed) Aconitase via Fenton reaction	^•^OH has a very short half-life and is very rapidly involved in other reactions Polyamines

Singlet oxygen (O_2_)	Nonphotosensitized mechanisms of O_2_ generation^∗∗^	O_2_ is rapidly implicated in many oxidation reactions Polyamines

Hydroperoxyl radical (HOO^•^)	Protonated form of O_2_^•−^	Nonenzymatic scavengers

Peroxyl radical (ROO^•^)	Polyunsaturated fatty acid metabolism	Nonenzymatic scavengers

Alkoxyl radical (RO^•^)	Polyunsaturated fatty acid metabolism	Nonenzymatic scavengers

^∗^Described in [150, 269]; ^∗∗^well reviewed in [270].
